# Sweet Pepper (*Capsicum annuum* L.) Canopy Photosynthesis Modeling Using 3D Plant Architecture and Light Ray-Tracing

**DOI:** 10.3389/fpls.2016.01321

**Published:** 2016-09-09

**Authors:** Jee Hoon Kim, Joon Woo Lee, Tae In Ahn, Jong Hwa Shin, Kyung Sub Park, Jung Eek Son

**Affiliations:** ^1^Department of Plant Science and Research Institute of Agriculture and Life Sciences, Seoul National UniversitySeoul, South Korea; ^2^Department of Horticulture and Breeding, Andong National UniversityAndong, South Korea; ^3^Protected Horticulture Research Institute, National Institute of Horticultural and Herbal ScienceHaman, South Korea

**Keywords:** FvCB model, light interception, paprika, photosynthetic rate, vertical position

## Abstract

Canopy photosynthesis has typically been estimated using mathematical models that have the following assumptions: the light interception inside the canopy exponentially declines with the canopy depth, and the photosynthetic capacity is affected by light interception as a result of acclimation. However, in actual situations, light interception in the canopy is quite heterogenous depending on environmental factors such as the location, microclimate, leaf area index, and canopy architecture. It is important to apply these factors in an analysis. The objective of the current study is to estimate the canopy photosynthesis of paprika (*Capsicum annuum* L.) with an analysis of by simulating the intercepted irradiation of the canopy using a 3D ray-tracing and photosynthetic capacity in each layer. By inputting the structural data of an actual plant, the 3D architecture of paprika was reconstructed using graphic software (Houdini FX, FX, Canada). The light curves and *A*/*C*_*i*_ curve of each layer were measured to parameterize the Farquhar, von Caemmerer, and Berry (FvCB) model. The difference in photosynthetic capacity within the canopy was observed. With the intercepted irradiation data and photosynthetic parameters of each layer, the values of an entire plant's photosynthesis rate were estimated by integrating the calculated photosynthesis rate at each layer. The estimated photosynthesis rate of an entire plant showed good agreement with the measured plant using a closed chamber for validation. From the results, this method was considered as a reliable tool to predict canopy photosynthesis using light interception, and can be extended to analyze the canopy photosynthesis in actual greenhouse conditions.

## Introduction

Canopy photosynthesis is one of the important factors for estimating crop growth and establishing the strategy of CO_2_ fertilization inside a greenhouse. Because crop yield is closely related to the seasonal integral of the total canopy photosynthesis, it can be used as base data to predict the crop production in a greenhouse (Monteith, 1965). Additionally, by estimating the attenuation of CO_2_ concentration with time, supply rates of CO_2_ fertilization in a cultivation system could be determined. In general, canopy photosynthesis is primarily determined by the light regime inside the greenhouse, and several factors such as meteorological and greenhouse structural factors, must be considered. Scaling up from the leaf to the canopy, the vertical pattern of the intercepted irradiation can be affected by the vertical structure of the whole plant and additional shading effects would occur from neighboring plants in the canopy (Caldwell et al., 1986; Chen et al., 1999). Other variances such as the direction of the sunlight, the ratio of the diffuse light, the greenhouse structure, the plant growth stage, and the plant density also affect the intercepted irradiation inside the canopy (Elifis et al., 1989; Lieth and Pasian, 1990; Stirling et al., 1994; Buck-Sorlin et al., 2011). Therefore, in estimating the canopy photosynthesis, it is important to investigate the light interception of the plant caused by these variances.

It is difficult to measure the actual light interception of the plant surface because of technical limitations. Therefore, previous research has estimated the canopy photosynthesis by various modeling approaches. Among the various approaches, the single leaf models, such as the FvCB model (Farquhar et al., 1980), represent the leaf level biochemical mechanism, and the whole canopy models, including the sunlit-shaded model (de Pury and Farquhar, 1997), are the most well-known models for photosynthesis (Zhu et al., 2012). Although these models are useful, they have been seldom used in greenhouse crop species (Gonzalez-Real and Baille, 2000; Yin and Struik, 2009; excepting cucumber Chen et al., 2014a; tomato de Visser et al., 2014; and rose Buck-Sorlin et al., 2011). Primary assumptions are that the absorbed photosynthetic active radiation affects the photosynthetic capacity of each canopy layer and contributes to the entire canopy photosynthesis (Field, 1983; de Pury and Farquhar, 1997; Roux et al., 1998; Dreccer et al., 2000; Johnson et al., 2010). Additionally, to simplify the calculation procedures, models have assumed that the vertical distribution of light interception has a negative exponential pattern from the top to the bottom of the canopy (Monsi and Saeki, 1953; Norman, 1980). The high level of spatial and temporal heterogeneity of the light interception is not considered in these models, for example, the shading by upper leaves on the lower part of the canopy, diffuse radiation which penetrates deep into the canopy (de Pury and Farquhar, 1997; Hikosaka, 2014), and the plant architecture affected by the leaf shape and angle in the light interception (Kim et al., 2010; Tang et al., 2015).

For analysis of the canopy photosynthesis rate, precise light distribution, and leaf photosynthesis are prerequisites (Chen et al., 2015). From this perspective, the construction of a 3D graphic plant is necessary to reflect the precise physical properties of the plant structure. Ray-tracing technique is a reasonable solution to incorporate optical properties such as the reflectance and transmittance of a leaf and other structures into the light simulation. Recently, there are increasing amount of studies where the light interceptions of crops have been estimated by using 3D plant models and light ray-tracing methods (Mabrouk et al., 1997; Buck-Sorlin et al., 2011; Sarlikioti et al., 2011; der Zande et al., 2011; Chen et al., 2014a,b; de Visser et al., 2014; Tang et al., 2015; Kang et al., 2016). To estimate canopy photosynthetic rates by combining above methods and photosynthetic models would be helpful for designing greenhouse crop production system. Thus, the objectives of the current study are to analyze accurate light interceptions using a 3D ray-tracing method, determine the vertical distributions of photosynthetic parameters, calculate the photosynthesis rate of each layer, and validate the canopy photosynthesis of paprika.

## Materials and methods

### Cultivation conditions

This experiment was conducted in a Venlo-type glasshouse located at the experimental farm of the Seoul National University in Suwon, Korea (37.3°N, 127°E). Paprika plants (*Capsicum annuum* L.) were transplanted after 3 months (20 July–15 October 2014) in rock wool cubes with a plant density of 3 plants/m^2^ and the distance between rows was 80 cm. Air conditioners were installed in each wall of the glasshouse to maintain a temperature between 25° and 35°C inside the greenhouse during the summer season and the relative humidity was controlled to be within a range of 60–80% using fogging systems. Nutrient solutions were irrigated 4 times a day at 10:00, 12:00, 14:00, and 16:00. To prevent a deficit of N related to the biosynthesis of chlorophylls, the total N concentration in the nutrient solution was NO_3_-N 1.45 mM. The other concentrations of macro-elements in the nutrient solution included P 1.61, K 3.59, Ca 4.00, Mg 1.88, and S 1.88 mM. The EC and pH ranges of the nutrient solutions were 2.6–3.0 dS m^−1^ and 5.5–6.5, respectively. The plants were pruned to form two main stems, which were vertically trellised to a “V” canopy system (Jovicich et al., 2004).

### Leaf photosynthesis and leaf nitrogen measurements

Eight layers were determined within each plant to investigate the vertical pattern of the leaf photosynthetic capacity. Each layer consists of four leaves and layer number was counted acropetally.

The leaf photosynthesis was measured with a portable photosynthesis system (LI-6400, LI-COR, USA). A closed chamber on the photosynthesis system was set at 25°C for the leaf temperature and 60–70% for the relative humidity to obtain the photosynthetic parameters on the standard temperature condition. Additionally, a red 8:blue 2 light quality of an LED light source similar to the sun spectrum was used inside the closed chamber. By using the auto program of the light curve and an *A*/*C*_*i*_ curve that measures 20 points for each program, photosynthesis were measured at (1) photosynthetic photon flux density (PPFD) = 1000 μmol m^−2^ s^−1^ under varying external CO_2_ partial pressure (*p*_*a*_ = 0–120 Pa) and (2) external CO_2_ partial pressure (Pa) = 100 Pa with varying PPFD (50–1000 μmol m^−2^ s^−1^). The calculated values of the internal CO_2_ partial pressure (*C*_*i*_) were provided by the LI-COR system inside the device.

After the photosynthesis measurement, each sample leaf was collected to determine the leaf nitrogen content. An average value of the leaf nitrogen content per layer was investigated after the Kjeldahl digestion of the leaves, which were oven-dried at 80°C for 5 days and then grounded (Kjeldahl, 1883).

### Entire-plant photosynthesis measurements

**Figure 1:**
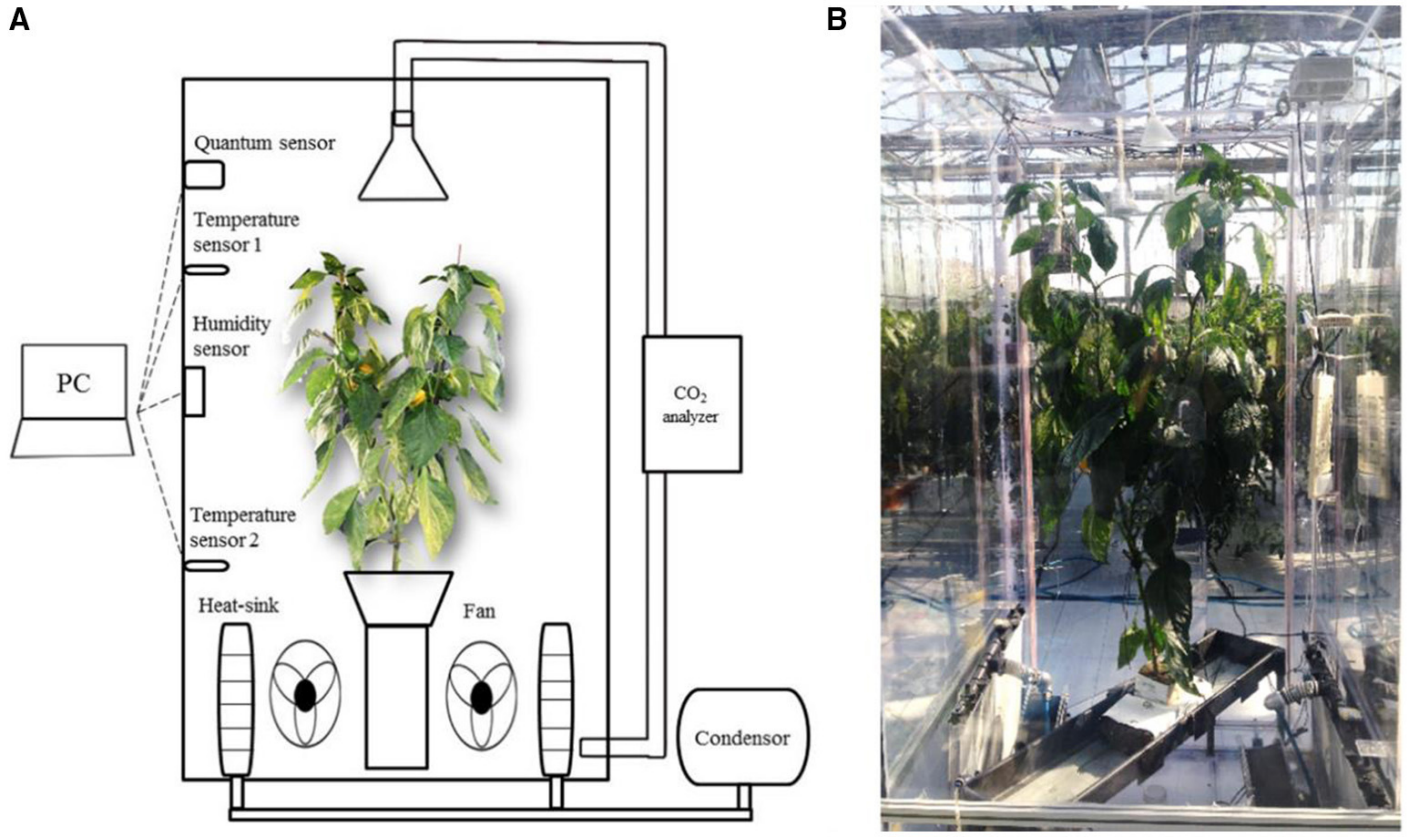
**A schematic diagram (A), and actual installation (B) of a closed growth chamber for measuring CO_**2**_ consumption of paprika plant**.

**Figure 2:**
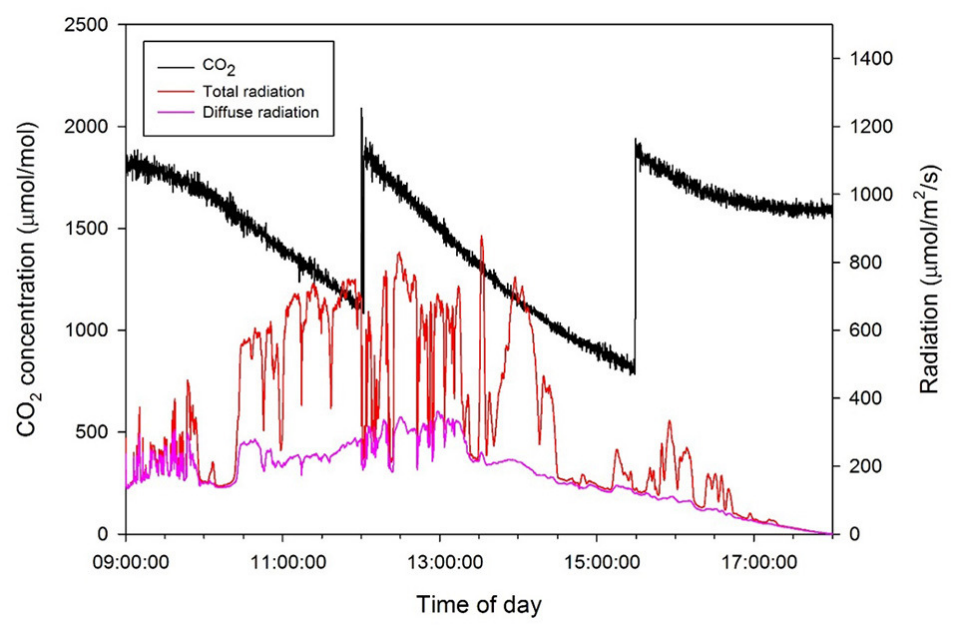
**Daily changes in CO_**2**_ concentration in the closed chamber with light intensity above the plant on 15 October 2014**.

To measure the daily CO_2_ consumption of an entire plant, a closed chamber (1 × 1 × 2 m) was designed and constructed using transparent polycarbonate. A closed chamber on the photosynthesis system was set at 25°C for the leaf temperature and 60–70% for the relative humidity (Figure 1); this is referred to as an open chamber system (Garcia et al., 1990). The CO_2_ concentration inside the chamber was set to range between 80 and 200 Pa to measure the photosynthesis rate of the entire plant while maintaining a CO_2_ level above the saturation points (Shin et al., 2011). An additional supply of CO_2_ gas was implemented when the CO_2_ concentration in the chamber was ~80 Pa. The CO_2_ concentrations inside the chamber were detected using a CO_2_ analyser (LI-820, LI-COR, USA). CO_2_ leakage of the chamber was about 0.2–0.3 μmol CO_2_ s^−1^ under the experimental CO_2_ condition (Figure S1). Irradiance inside the chamber was measured using an irradiation sensor (BF5, Delta-T Devices, UK) and the diffuse ratio was also determined. To maintain the temperature and CO_2_ concentration inside the chamber, two radiators circulating cool water were placed along each sidewall. A fan was passed through the radiators and blown toward the chamber wall to maintain equal ventilation. The temperature inside the chamber was maintained at 25°C by circulating cooled water controlled by a condenser (DH-003A, Daeho-condenser, Korea). The CO_2_ concentration, irradiance, and temperature inside the chamber were stored in a data logger every 10 s. Silica gel was used in the air circulation process to control increased humidity from the transpiration of the plant. A plant was selected from among five samples and was placed in the chamber from 9:00 to 18:00. Whenever the CO_2_ concentration reached approximately 100 Pa, additional CO_2_ was supplied to retain a saturated CO_2_ condition (Figure 2).

### Construction of the 3D virtual plant

Before sealing the chamber for measuring the entire plant's photosynthesis, a sample plant free from disorders was chosen to design the 3D virtual plant. The structure of the sample plant was measured using a ruler and protractor to transpose the real structure of the plant to a 3D graphic. The architect parameters consisted of three major parts (leaf, petiole, and stem) and the detailed measurements included the following: (1) leaf area and leaf angle; (2) petiole length and petiole angle; and (3) stem length, stem diameter, and stem angle. The area of each leaf within the sample plant was measured using a leaf area meter (LI-3100, LI-COR, USA). Structural characteristics of leaves and stems by layer were measured as Table 1.

**Table 1:** **Structural characteristics of leaves and stems by layer**.

**Layer**	**Leaf**				**Stem**		
	**Leaf area (cm^2^)**	**Petiole length (cm)**	**Dropness (°)**	***n*^z^**	**Radius (mm)**	**Length (cm)**	***n*^z^**
1	181.4	7.0	30.33	4	4.3	8.3	2
3	189.9	6.3	37.08	4	4.5	7.5	2
5	200.3	5.3	34.92	4	4.1	9.2	2
7	220.0	6.1	29.25	4	4.2	8.8	2
9	200.2	5.0	45.72	4	4.0	7.9	2
11	269.8	5.4	36.18	4	4.4	9.0	2
13	243.8	5.1	57.78	4	4.4	8.5	2
15	181.1	3.8	51.84	6	4.2	3.4	4

z *replicates*.

**Figure 3:**
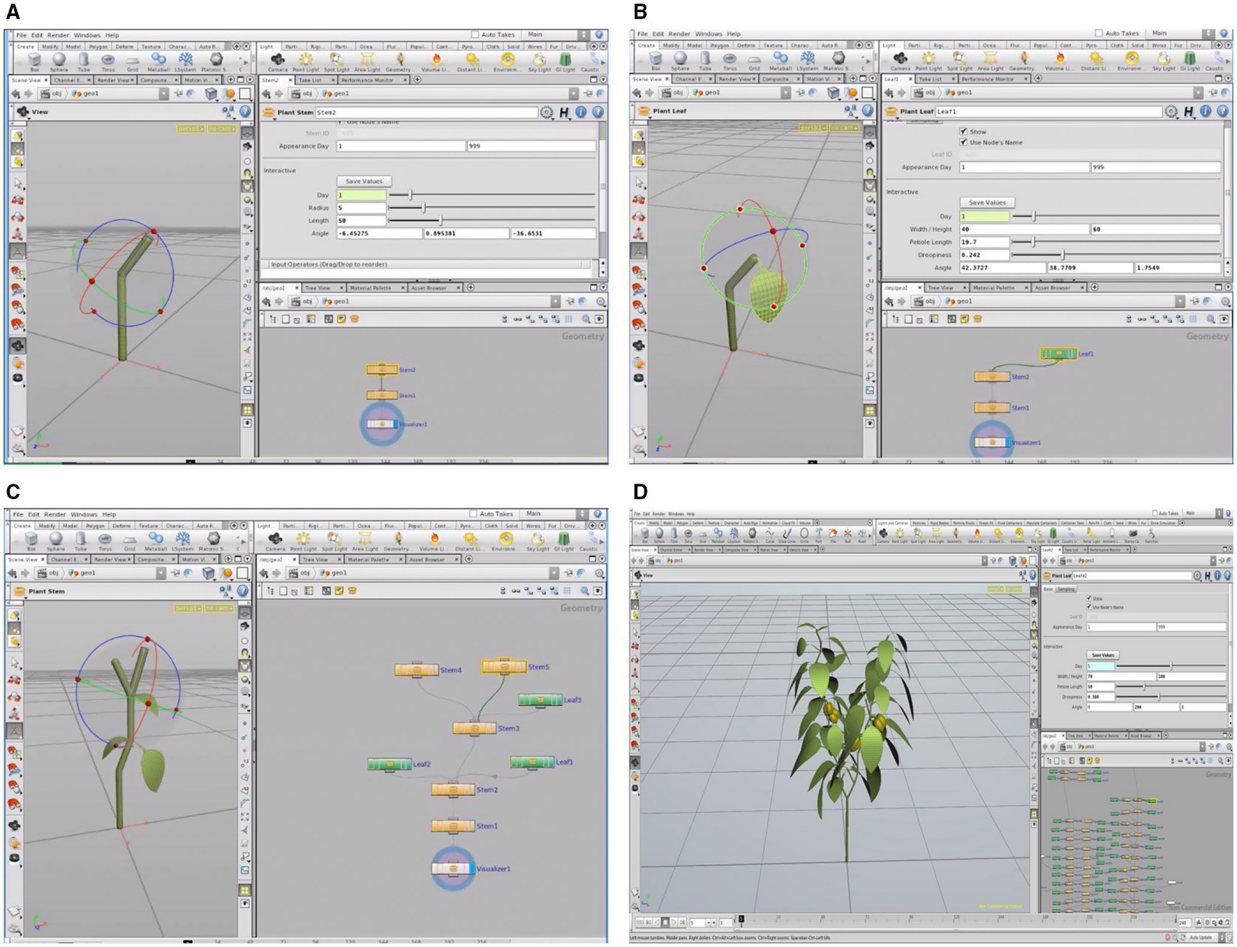
**A 3D virtual plant constructed in the L-system using the Houdini FX graphic software: construction of paprika stem (A) and leaf (B), the process of modeling the paprika (C), and tree window of L-system formalism and graphic window of the completed paprika structure (D)**.

A 3D plant model was developed using graphic software (Houdini FX, FX, Canada), as shown in Figure 3. Using an L-system formalism, which is useful in the construction of a plant's growth pattern, the plant structure could be built up from the bottom to the top in the tree window (Figure 3D) by applying structure values for each part of the plant. For the validation procedure, the actual plant inside the closed chamber was virtualized as a 3D graphic plant that referred to the measured values of the plant structure and the digitized data using a 3D digitizer (Sense, 3D systems, Inc., USA). The virtual plant consisted of two primary stems having 15 nodes each. The calculation of the leaf area (LA) is determined using the length (L) and width (W). The leaf area equation is embedded inside the graphic tool, LA = 0.6034 LW + 0.0732 (*R*^2^ = 0.994, *p* < 0.001; Tai et al., 2009). The leaves with an accurate leaf area were simultaneously shown on the graphic window when the users input the values of L and W (Figure 3D). The petiole and leaf angles were also applied by inputting the angles (x, y, z) of the directions (Figures 3A,B).

### Simulation of the intercepted irradiation

**Figure 4:**
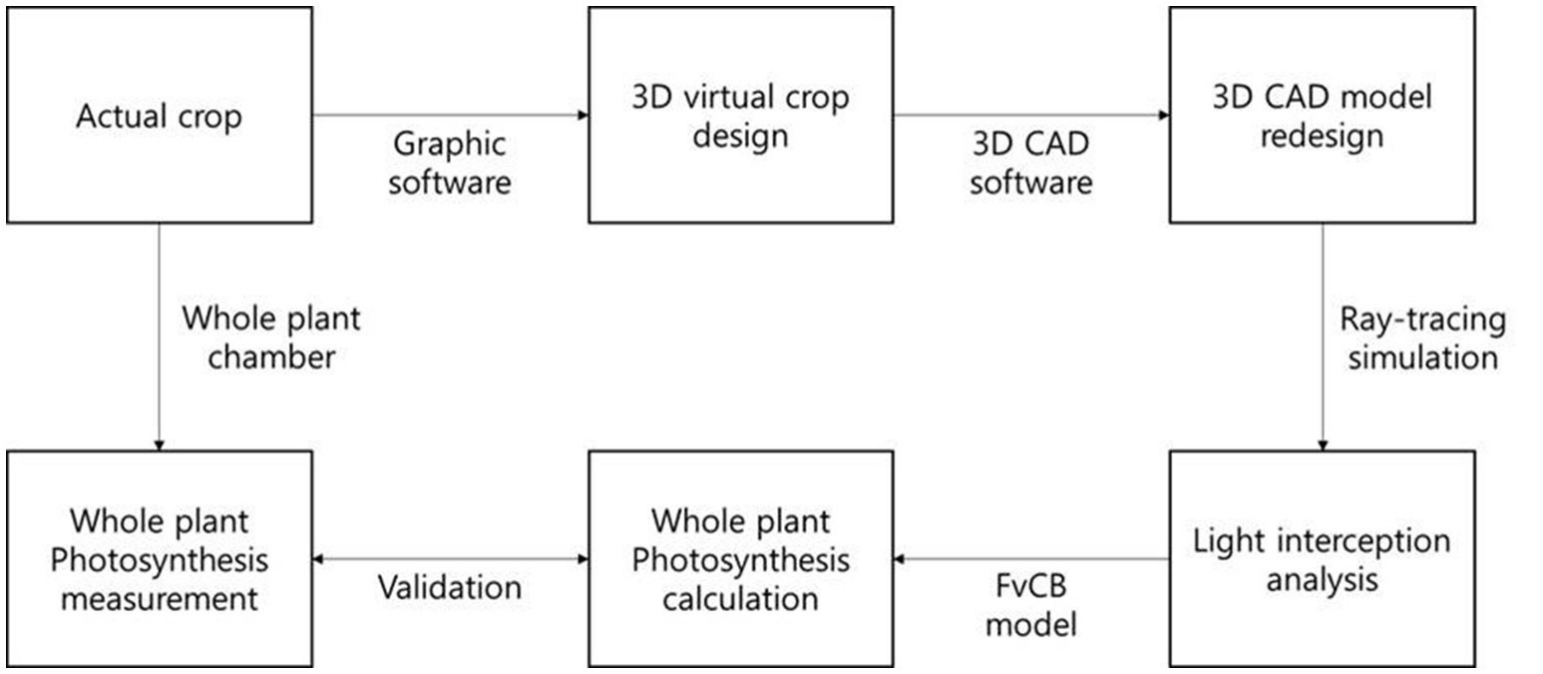
**A work flow for construction of 3D plant model, calculation, and validation of a whole plant photosynthesis rate**.

Redesigning the 3D plant was accomplished using 3D CAD software (SOLIDWORKS, Dassault Systemes, FRANCE), and light interception analysis was simulated using ray-tracing software (OPTISWORKS, OPTIS Inc., FRANCE). Light illuminance on the surface of the leaves of the 3D plant model was calculated to investigate the intercepted irradiance in specific conditions and values of light intensity were obtained on the 3D leaf surface. The growth chamber was modeled with the 3D CAD software and assembled with the 3D plant model. With the simulation software it was possible to input microclimate parameters: sun directions (coordinates, date, time, zenith, north direction), and sunlight properties (ratio of direct light and diffuse light); and material parameters: optical properties of the leaf, chamber, and glasshouse structure. Optical properties (transmittance and reflectance) were measured using an integrating sphere (IC2, StellarNet Inc., CANADA) with a spectrometer (BLUE-Wave, StellarNet Inc., CANADA) and a light source (SL1 Tungsten Halogen, StellarNet Inc., CANADA) and entered in the preferences section for the leaves in the simulation program (Figure 4). In the leaf optical measurements, the optical properties of the leaves have little differences in the vertical position within the plant. The reflectance and transmittance of both sides of leaf were used as 0.1 and 0.07, respectively, in our simulation; Ray-tracing simulations were conducted with 10 giga rays and the number of max impacts was set to be 10 for all conditions. Identifying the applicability for expanding to a canopy situation, plant arrays of 1 × 1 and 3 × 3 with a distance of 0.8 m between plants were set to investigate the different patterns of intercepted radiation. Detectors were placed on the surface of a single plant located in the center of the canopy. Four cases were simulated at 9:00, 12:00, 15:00, and 18:00 and the intercepted irradiance was analyzed for each layer.

### Calculation of the photosynthetic parameters

*A*/*C*_*i*_ curve fitting utility was used to calculate the photosynthetic parameters (Sharkey et al., 2007). The prediction of the leaf photosynthesis rate was based on the FvCB model (Equation 1).

(1) $$\begin{array}{r} A_{l}=\min\left\{\begin{array}{c} A_{v},f\left(V_{lm},c_{i},T_{l}\right)\\ A_{j},f\left(I,f,J_{m},c_{i},T_{l}\right) \end{array}\;\right\}-R_{l},f\left(T_{l}\right) \end{array}$$

where *A*_*l*_ is the rate of the leaf's net assimilation, and *A*_*v*_ and *A*_*j*_ indicate the rates of the leaf gross assimilation limited by ribulose biphosphate-carboxylase-oxygenase (Rubisco) activity and ribulose biphosphate (RuBP) regeneration, respectively. *A*_*l*_ is determined by the minimum value of the two rates (Equation 1). Each rate can be expressed as various leaf characteristics (the maximum photosynthetic Rubisco capacity, *V*_*lm*_, and the maximum rate of electron transport, *J*_*m*_). All of the temperature conditions in the current experiment were fixed at 25°C to neglect the effect of temperature on the parameters and, therefore, on the temperature-related functions in the model.

The maximum photosynthesis rate (*A*_*max*_) was calculated from the light curve at each layer by using a non-rectangular hyperbolic function. The photosynthetic Rubisco capacity (*V*_*l*_) was also obtained from the *A*/*C*_*i*_ curve by using a non-linear regression. Assuming that the CO_2_ fixation rate is limited only by Rubisco activity in a low CO_2_ condition, and *V*_*l*_ value of each layer was estimated from the *A*/*C*_*i*_ curve of each layer within the range *C*_*i*_ < 30 Pa (Equation 2). Similarly, the potential rate of the electron transport (*J*_*m*_) values was determined from the *A*/*C*_*i*_ curve at a range above 40 Pa for *C*_*i*_ (Equations 3, 4).

(2) $$\begin{array}{r} V_{l}=A_{v}\frac{\left(c_{i}+K\prime \right)}{\left(c_{i}-\Gamma_{*}\right)} \end{array}$$

(3) $$\begin{array}{r} J=4A_{j}\frac{\left(c_{i}+2\Gamma_{*}\right)}{\left(c_{i}-\Gamma_{*}\right)} \end{array}$$

(4) $$\begin{array}{r} J_{m}=J\frac{\left(I_{le}-\theta_{\text{l}}J\right)}{\left(I_{le}-J\right)} \end{array}$$

where *I*_*le*_ is the photosynthetic active radiation (PAR) effectively absorbed by PSII, and *J* is the rate of electron transport. Detailed model equations and constants are shown in Tables 2, 3. *I*_*l*_ is the total absorbed PAR per unit leaf area. Calculation of all the parameters were followed by de Pury and Farquhar (1997) and Kim and Lieth (2003).

**Table 2:** **Equations of the FvCB model**.

**Equation**	**Description**	**Number**
$A_{\text{l}}=\min\left\{A_{v},\;A_{j}\right\}-R_{l}$	Rate of leaf net photosynthesis	(A1)
$A_{v}=V_{l}\frac{\left(c_{i}-\Gamma *\right)}{\left(c_{i}+K^{\prime }\right)}$	Rubisco-limited photosynthesis	(A2)
$K^{\prime }=K_{c}\left(1+\frac{O}{K_{o}}\right)$	Effective Michaelis-Menten constant	(A3)
$A_{j}=J\frac{\left(c_{i}-\Gamma *\right)}{4(c_{i}+2\Gamma *)}$	Electron-transport limited rate of photosynthesis	(A4)
$\theta_{\text{l}}J^{2}-\left(I_{le}+J_{m}\right)J+I_{le}J_{m}=0$	Irradiance dependence of electron transport	(A5)
$I_{le}=\frac{I_{l}\left(1-f\right)}{2}$	PAR effectively absorbed by PSII	(A6)
$\frac{R_{l}}{V_{l}}=\frac{\Gamma -\Gamma *}{\Gamma +K^{\prime }}$	Ratio of leaf respiration to photosynthetic Rubisco capacity	(A7)

*Temperature condition, 25°C; other temperature-related functions are omitted.

**Table 3:** **Photosynthetic parameters and constants of the FvCB model at 25°C**.

**Symbol**	**Value**	**Unit**	**Description**
*K*_*c*_	40.4	Pa	Michaelis-Menten constant of Rubisco for CO_2_
*K*_*o*_	24.8 × 10^3^	Pa	Michaelis-Menten constant of Rubisco for O_2_
*O*	20.5 × 10^3^	Pa	Oxygen partial pressure
*R_lo_*	0.0089*V_lo_*	μmol m^−2^ s^−1^	Dark leaf respiration rate
Γ	4.4	Pa	CO_2_ compensation point of photosynthesis
Γ^*^	3.69	Pa	Γ in the absence of mitochondrial respiration
*f*	0.15	–	Spectral correction factor
θ*_l_*	0.68–0.83	–	Curvature of leaf response of electron transport to irradiance

* *Values of the photosynthetic parameters are given at 25°C*.

### Validation of whole plant photosynthetic rate

With an average value of intercepted irradiation from the simulation and photosynthetic parameters from the measurements at each layer, the leaf photosynthesis rate was calculated for each layer. To apply the actual light intensity from the logged data to an estimate, the average values of light intensity and the diffuse ratio for 30 min were used. By integrating the photosynthesis rate at each layer, the photosynthesis rate of the entire plant was calculated using the sum of *A*_*l*_ in each layer.

According to this method, the estimated data were calculated every half hour and these data of the entire plant's photosynthesis rate were compared with actual data from 9:00 to 18:00 on October 15, 2014, for validation. From an actual measurement of the CO_2_ concentration in a sealed chamber, the reduction of the CO_2_ concentration was converted to a photosynthesis rate assuming that the slope of the CO_2_ concentration is the same as the photosynthesis rate. A work flow for construction of 3D plant model, calculation, and validation of a whole plant photosynthesis rate was described as Figure 4.

## Results

### Distribution of the maximum photosynthesis rate and leaf nitrogen content within the entire plant

**Figure 5:**
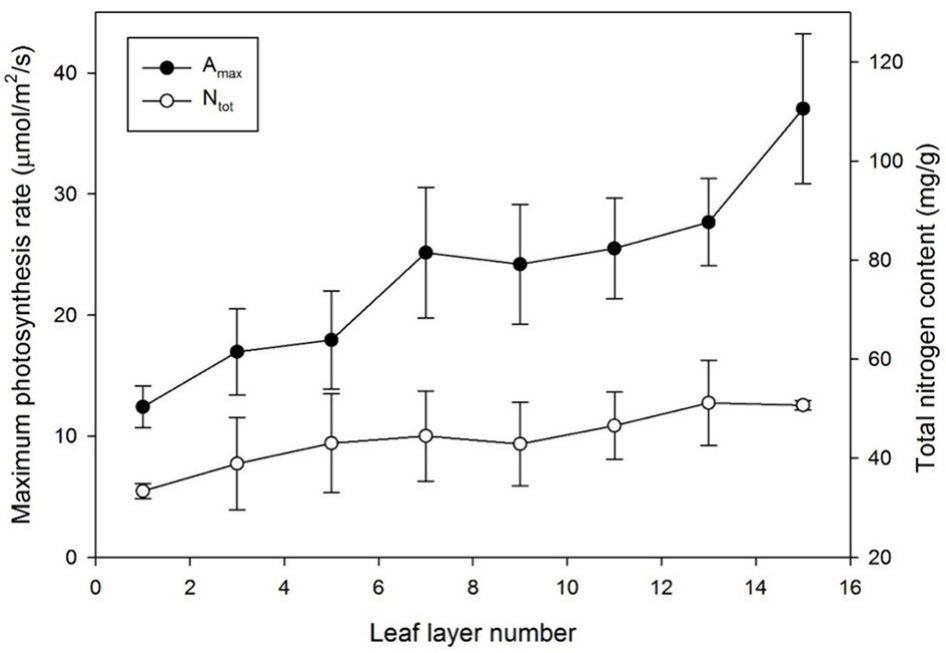
**Maximum photosynthesis rate (***A***_***max***_) and total nitrogen content (***N***_***tot***_) by leaf layer number**. Vertical bars represent the Mean ± *SE* (*n* = 5).

The maximum photosynthesis rate, *A*_*max*_, at each layer was measured to be within 1000 μmol m^−2^ s^−1^ of the light intensity and 100 Pa of the CO_2_ saturation condition as shown in Figure 5. The mean values of *A*_*max*_ decreased from the top (layer 15) to the bottom (layer 1), from 37.04 to 12.41 μmol m^−2^ s^−1^, respectively. The standard variations of *A*_*max*_ were somewhat higher in the upper part than the bottom, indicating that the range of *A*_*max*_ appeared broader in the younger leaves compared to the older leaves at the bottom. Unlike the exponential patterns generally assumed in many photosynthesis models, the distribution of *A*_*max*_ for an individual plant showed a linear pattern on all of the five sample plants.

In the case of nitrogen distribution, the total nitrogen content in each leaf increased with an increase in the leaf layer number. Although the standard deviation in the middle layer was greater, the total nitrogen content in the uppermost layer was more than double that in the bottom layer, similar to *A*_*max*_, and it was apparent that most of the nitrogen was allocated to the upper layer, which retained higher light use efficiency (Figure 5). Decreasing patterns of *A*_*max*_ and *N*_*tot*_ were very similar in the vertical distribution, indicating that the nitrogen content is strongly correlated with the photosynthetic capacity. Converting a leaf nitrogen content on a dry mass basis (mg g^−1^) to a leaf nitrogen concentration on a leaf area basis (g m^−2^), the spatial distribution of the nitrogen content on a leaf area basis was not significantly meaningful.

### Distribution of photosynthetic parameters, *V*_*lo*_ and *J*_*mo*_, within an entire plant

Comparing the light curves and *A*/*C*_*i*_ curves at each layer, the photosynthetic capacity in a certain position varied considerably among the layers, resulting in different light use efficiencies. Changes in *V*_*lo*_ and *J*_*mo*_ were identified by the leaf position using the measured value of *A*_*l*_ for each layer at 25°C (Table 4). The range of θ is a between 0.68 and 0.83 regardless of the leaf layer number. By increasing the leaf layer number, both the average values of *V*_*lo*_ and *J*_*mo*_ decreased from 88.62 and 175.42 (layer 15) to 20.31 and 50.83 μmol m^−2^ s^−1^ (layer 1), respectively. Despite significant variations, both average values of the parameters showed linear patterns in the vertical distribution rather than exponential patterns within the plant, similar to *A*_*max*_ and *N*_*tot*_. Average values of photosynthetic parameters were selected to use in the calculation of the photosynthesis rate at each layer.

**Table 4:** **Estimation of the photosynthetic parameters ***V***_***lo***_ (= value of ***V***_***l***_ at 25°C) and ***J***_***mo***_ (= value of ***J***_***m***_ at 25°C)**.

**Layer**	***V*_lo_****	***n*^y^**	***R*^2^**	***J_mo_***	***n*^y^**	***R*^2^**
15	88.62 ± 8.20^z^	5	0.76	175.42 ± 13.17	5	0.71
13	81.72 ± 5.74	5	0.83	123.97 ± 9.77	5	0.78
11	77.93 ± 6.24	5	0.70	119.64 ± 9.87	5	0.85
9	71.25 ± 5.60	5	0.80	107.64 ± 11.84	5	0.75
7	68.56 ± 6.88	5	0.69	88.63 ± 12.47	5	0.69
5	56.08 ± 7.09	5	0.73	72.62 ± 14.08	5	0.73
3	32.82 ± 7.70	5	0.66	67.49 ± 14.66	5	0.67
1	20.31 ± 2.57	5	0.88	50.83 ± 10.69	5	0.73

z *Mean ± SE*.

y *replicates*.

*n and R^2^ are the number of observed A/C_i_ curves per layer and the coefficient of determination by fitting non-rectangular hyperbolae functions, respectively*.

### Validation of an entire plant's photosynthesis and expansion to the canopy situation

**Figure 6:**
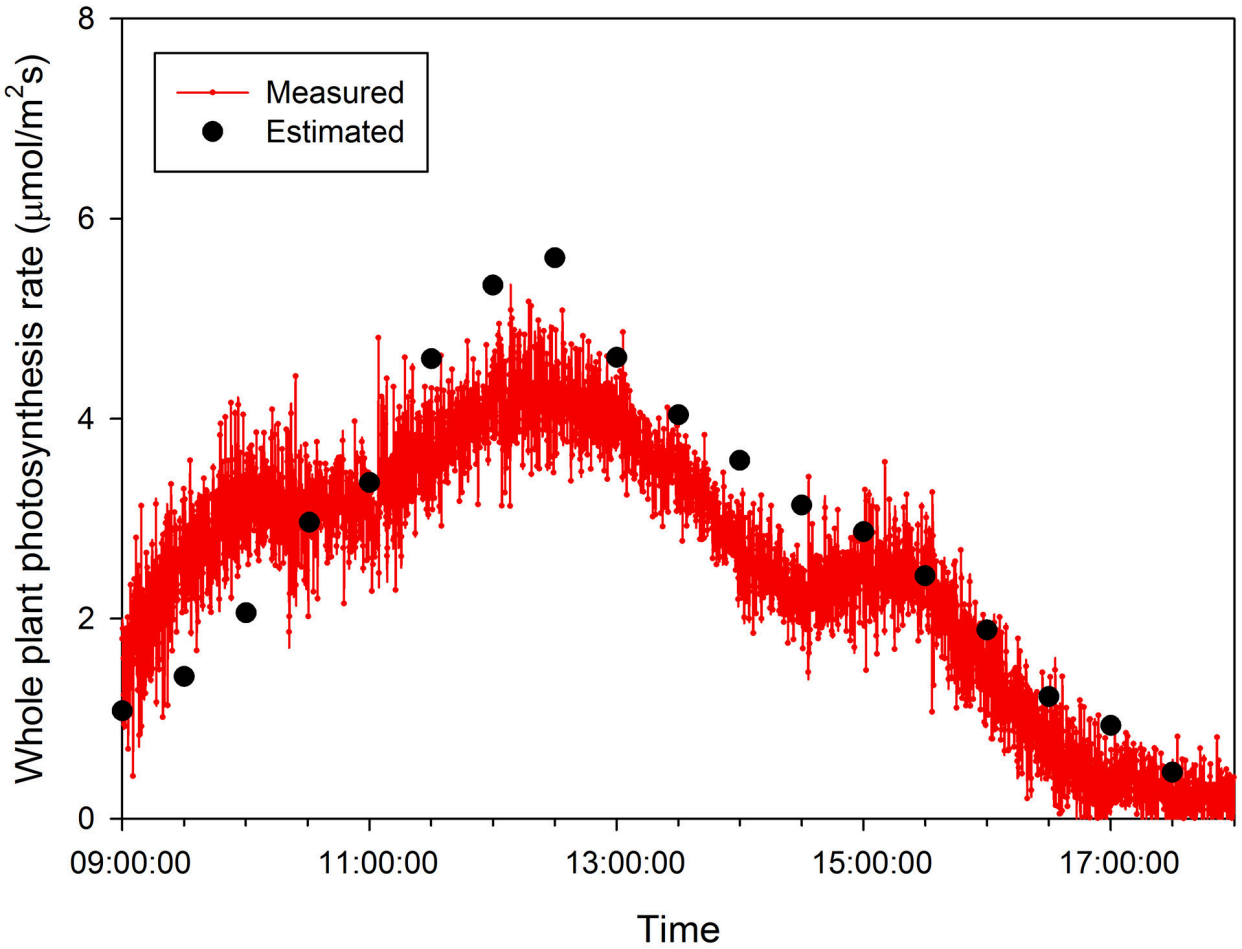
**A comparison of measured and estimated photosynthesis rates of the entire plant on 15 October 2014**.

**Figure 7:**
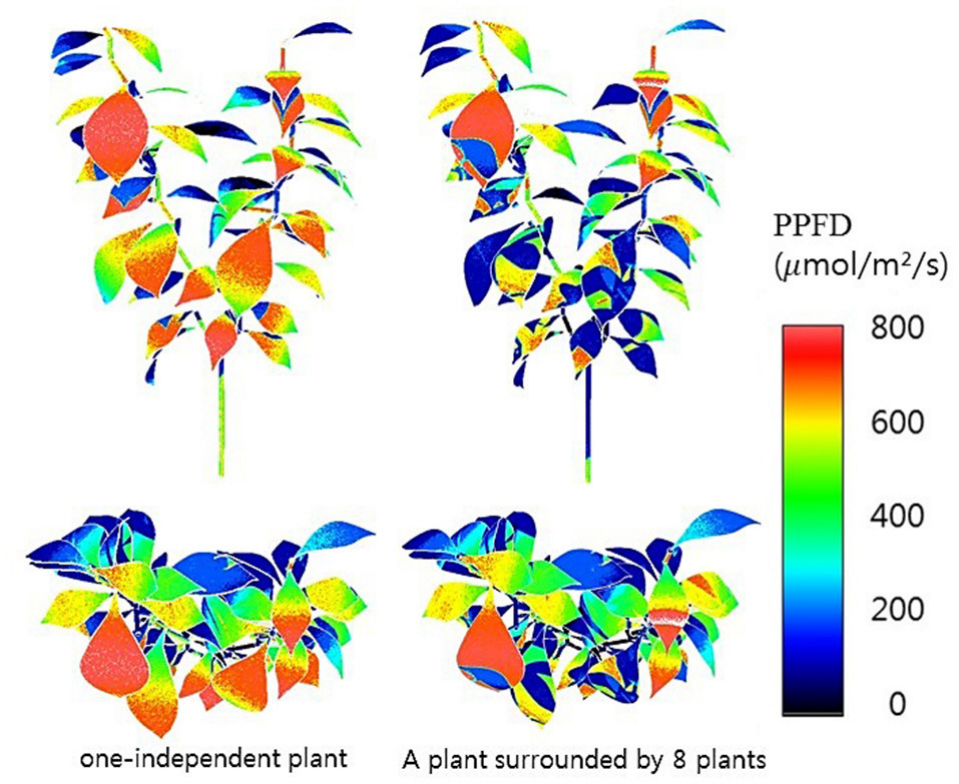
**3D simulated results of intercepted irradiances of a single plant at 12:00 not surrounded (left) and surrounded (right) by eight plants**.

**Figure 8:**
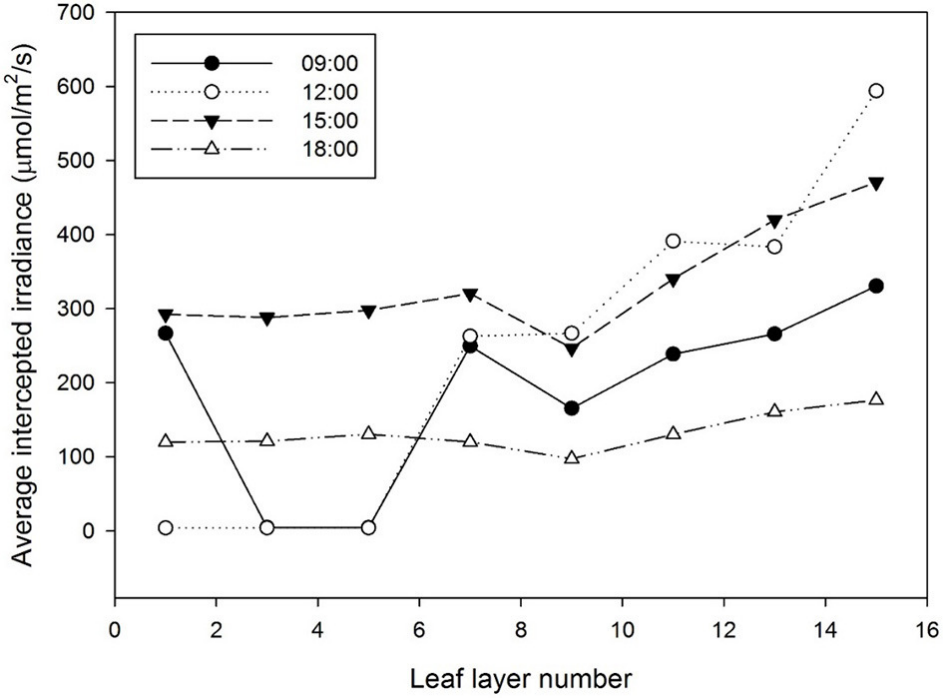
**Estimation of the average intercepted irradiation by leaf layer number from the ray-tracing simulation**. The detected sample was a center plant inside a 3 × 3 canopy cultivation condition.

Half hour-photosynthesis rates of the sample plant were compared with estimated rates and showed good agreement with a coefficient of determination (*R*^2^) of 0.85 and a root mean square error (RMSE) of 0.47 (Figure 6). The estimation values were slightly lower than actual values at 9:00 to 10:30 and upper at 11:30 to 15:00. Daily variations in the photosynthesis rates were clearly shown in the estimated data. For the canopy situation, the 3D simulated data explicitly shows the shading effect of the neighboring plants, which mostly appeared in the middle and bottom layers (Figure 7). From an overhead view the intercepted irradiance within the plant was primarily affected by the plant, which was oriented toward the sun. The total intercepted radiation of the center plant surrounded by eight plants did not decrease significantly regardless of the number of neighboring plants and the shade time determined by the height of the neighboring plants. In estimating the intercepted irradiation at each layer, linear decay appeared at the top and middle layers, and the variations in the intercepted irradiation occurred as a result of the changes in sun direction (Figure 8).

## Discussion

Canopy photosynthesis is such a complex mechanism that the correlation of various environmental factors engaged in the photosynthesis process should be considered. Furthermore, scaling up from leaf to canopy, intercepted radiation and the optical and physiological properties related to the photosynthetic capacity should be considered, including leaf age, leaf acclimation to light, and nitrogen distribution within the canopy. However, it is necessary to clarify the relationship between light acclimation and nitrogen distribution, which is also heterogeneous within the canopy. Intercepted radiation is sensitively affected by leaf shape, leaf angle, and plant position inside the canopy architecture (Gonzalez-Real and Baille, 2000; Sarlikioti et al., 2011); therefore, precise measurement of intercepted radiation on the plant surface is not easy because of technical limitations.

Accordingly, 3D model is necessary for analysis of intercepted irradiation (Figure 3). To obtain the precise architecture for a plant in a 3D model, advanced technology for scanning the material is required to simulate the intercepted radiation on the plant surface, and the supporting hardware should be accompanied by an increased number of rays in the optical simulation. In actual canopy conditions, the heterogeneity of plant architecture still exists such that the compromise between precision and simplicity is inevitable in analyzing canopy photosynthesis. A simplified analysis method with guarantee of its accuracy is strongly required to perceive dynamic changes of the canopy photosynthesis rate in real-time.

There are still many possibilities to improve the analysis by (1) parameterization by using chlorophyll fluorescence as input (Bellasio et al., 2016), (2) development of dynamic architectural models related with physiological events (Chen et al., 2014b, 2015), (3) analysis of horticultural practices on the model behavior (de Visser et al., 2014), (4) specification of the relationships between nitrogen content and photosynthetic parameters, (5) analysis of photosynthesis as influenced by stomatal conductance and CO_2_ concentration (Kim and Lieth, 2003), and (6) reduction of CO_2_ leakage and inhomogeneous air mixing in the chamber.

Especially, the spatial distribution of nitrogen allocation provides another method for estimating canopy photosynthesis with regards to the photosynthetic capacity. Standard deviations of the nitrogen content exist primarily in the middle layers, indicating that the heterogeneity of the light environment in the canopy primarily occurred as a result of additional influences such as a sun fleck or shading effect by neighboring plants. Although many researchers determined the optimal distribution of nitrogen in nature to be a method for maximizing canopy photosynthesis (Hirose and Werger, 1987; Schieving et al., 1992), the detailed mechanism of leaf acclimation to the light environment remains under investigation including the quantitative analyzes of photosynthetic capacity (Ellsworth and Reich, 1993; Iio et al., 2005; Anten and During, 2011). The spatial distribution of nitrogen within the canopy is greatly simplified by models to calculate the canopy photosynthesis, and fixing the pattern of the photosynthetic parameters may cause errors in the precise estimation of canopy photosynthesis. In the current study, the leaf layer criteria of paprika were simplistically established because of the plant architecture pattern. However, additional criteria might be required for other species such as leafy vegetables that have a horizontal structure rather than a vertical structure.

In addition, other environmental factors such as the external CO_2_ concentration, temperature, and leaf age also affect the leaf properties of photosynthesis (Thornley, 2002; Escudero and Mediavilla, 2003). It was assumed in this research that the spatial distributions of the external CO_2_ concentration and temperature are identical within the canopy. Paprika transplanted after 3 months were chosen in the current analysis because of the closed chamber's size limitations. In applying later growth stages, the conditions for the analysis should be modified because the intercepted radiation is changed by neighboring plants whose heights are greater. Furthermore, leaf age functions should be incorporated into the FvCB model (Irving and Robinson, 2006) to estimate the photosynthesis of each leaf for different growth stages within the canopy.

By measuring the actual photosynthetic rates of a whole plant in the growth chamber, we could validate the canopy photosynthetic results estimated with the 3D plant model and light ray-tracing method. Interestingly, the measured canopy photosynthesis (Figure 6) did not simultaneously reflect the fluctuating light condition (Figure 2). This might be due to that, technically, the air in the chamber could not be homogeneously mixed. Estimated values were around 5–10% different from measured. In spite of several existing limitations in estimating the canopy's photosynthesis, the simulation method developed is quite suitable for precisely predicting the canopy's photosynthesis rate by applying microclimatic factors such as the location, date, time, diffuse ratio, and optical properties of the materials. From the simulated data, the sun fleck inside the canopy was found to change with time and the average light intensity of a certain layer depends on the amount of direct sunlight. Furthermore, fractions of sunlit and shaded areas changed through time (Figure 2), with the result that the vertical distributions of intercepted radiation did not always follow the pattern of the Lambert-Beer's law that irradiation is exponentially decayed (Figure 8). An increase in the diffuse ratio of sunlight reduces light variations among the leaves at different locations in the canopy. The current simulation results identified that the pattern of intercepted irradiation within the entire plant was strongly determined by sun direction and its optical properties.

In the current study, an analysis method to determine the canopy photosynthesis was developed using graphic software based on a 3D model and ray-tracing simulation (Figure 7). The photosynthetic capacity within the plant was significantly different among the vertical positions (Table 4). With the intercepted irradiation data and photosynthetic parameters of each layer, the values of an entire plant's photosynthesis rate were estimated by integrating the calculated photosynthesis rate at each layer. The estimated photosynthesis rate of an entire plant showed good agreement with the measured plant using a closed chamber for validation (Figure 6). The advantages of this method are the availability of precise analysis of canopy photosynthesis considering various environmental factors and the expendability to a greenhouse cultivation condition. By expanding this approach to canopy conditions, it is possible to analyze the canopy's photosynthesis as a key factor in a cultivation system. By supplementing plant physiological aspects, the method could be a powerful tool to predict the mass production of horticultural crops in greenhouses.

## Author contributions

JK and JL developed the 3D model, conducted the measurement and simulation. TA, JHS, and KP constructed the growth chamber and controlled the environments. JES designed the experiment, developed the 3D model, and conducted the total analysis.

### Conflict of interest statement

The authors declare that the research was conducted in the absence of any commercial or financial relationships that could be construed as a potential conflict of interest.

## Supplementary material

Figure S1 **Change in CO_**2**_ concentration in the closed chamber without plant for 9 h**.
