# Supplementary figures and images for: Sweet Pepper (Capsicum annuum L.) Canopy Photosynthesis Modeling Using 3D Plant Architecture and Light Ray-Tracing
Source: Front Plant Sci. 2016 Sep 9;7:1321. doi: 10.3389/fpls.2016.01321 (PMC5016622; doi:10.3389/fpls.2016.01321)

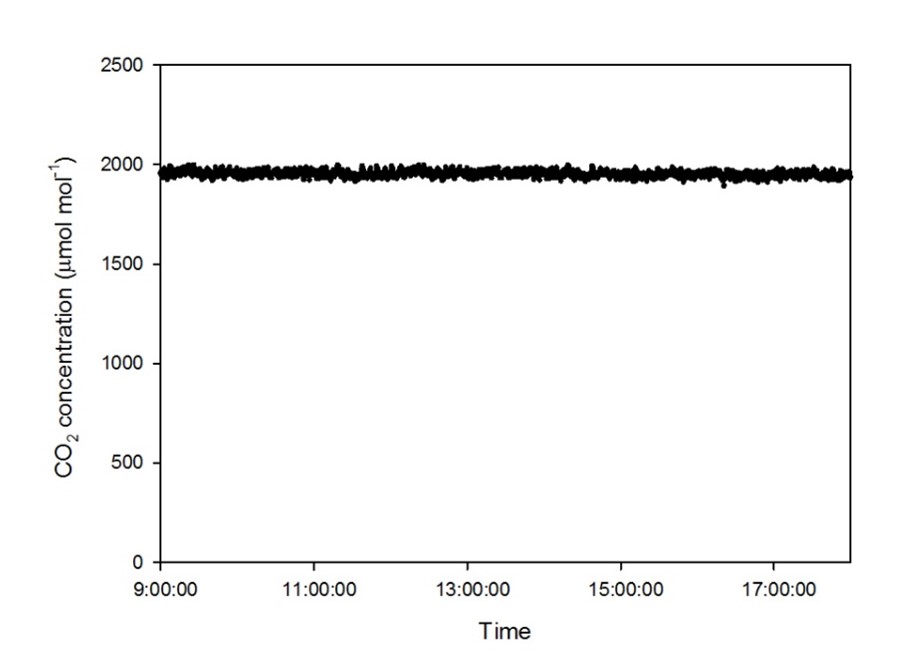

Supplement: Figure S1 — Change in CO2 concentration in the closed chamber without plant for 9 h. [file Image1.JPEG]
